# Validation of the Mental Health Literacy Scale in French University Students

**DOI:** 10.3390/bs12080259

**Published:** 2022-07-28

**Authors:** Ilaria Montagni, Juan Luis González Caballero

**Affiliations:** 1 Bordeaux Population Health Research Center UMRS1219, University of Bordeaux, Inserm, F-33000 Bordeaux, France; 2 Department of Statistics and Operational Research, University of Cádiz, 11003 Cádiz, Spain; juanluis.gonzalez@uca.es

**Keywords:** measure, knowledge, psychometric analyses, validation, mental health literacy, students

## Abstract

Background: Mental health literacy is a determinant of mental health, which can facilitate early detection of psychological problems and endorse timely access to care. Instruments to measure mental health literacy exist, but not in French. Assessment of mental health literacy in young adults is essential to tailor appropriate educational interventions promoting psychological wellbeing and preventing mental health problems in this vulnerable population. The aim of this study was to validate the French version of the Mental Health Literacy Scale (MHLS-FR) in university students. Methods: A total of 482 students from the University of Bordeaux, France, completed the translated version of the scale. Collected data were used to validate the MHLS-FR through psychometric analyses: descriptive statistics, item distribution, test-retest reliability, exploratory structural equation model, confirmatory factor analysis, Cronbach’s alpha and McDonald’s omega coefficients, and hypothesis testing. Results: The final scale included 26 items covering 6 dimensions. Cronbach’s alpha and McDonald’s omega coefficients were 0.744 and 0.961, respectively. With test-retest, about 50% of items had intraclass correlation coefficients superior to 0.5. Conclusions: The MHLS-FR can be considered as a valid and reliable instrument for measuring mental health literacy in French students.

## 1. Introduction

Mental health literacy (MHL) was first defined as “knowledge and beliefs about mental disorders which aid their recognition, management or prevention” [1] and consisted of six domains: “ability to recognise specific disorders or different types of psychological distress”; “knowledge and beliefs about risk factors and causes”; “knowledge and beliefs about self-help interventions”; “knowledge and beliefs about professional help available”; “attitudes which facilitate recognition and appropriate help-seeking”; and “knowledge of how to seek mental health information” [2]. MHL is a determinant of mental health that can facilitate the early detection of psychological problems and promote timely access to care [3]. The underpinning assumption is that mental health knowledge can improve mental health per se. Improving MHL empowers people by helping them develop skills they need for their psychological wellbeing such as positive attitudes and good mental health care decisions. Therefore, it is crucial to increase MHL levels across populations.

### 1.1. Measuring Mental Health Literacy

Measuring instruments in public health have three objectives: provide baseline information for monitoring patterns of health; provide information for planning, developing, and evaluating an intervention; and raise awareness and improve the agenda-setting for health promotion [4]. The unique instrument that captures all dimensions of MHL is the Mental Health Literacy Scale (MHLS) [5]. It consists of 35 items, which are grouped so as to cover the six dimensions identified by Jorm et al. [1]. The MHLS is a methodologically and psychometrically reliable measurement [6,7]. It has been conceptualized and validated in Australia in a large population (*n* = 617) including university students. Performed analyses have demonstrated its construct validity, internal consistency, and test-retest reliability. The MHLS has been translated and validated in several languages. A translated scale should measure the same construct (MHL) in exactly the same way across languages and countries through functional equivalence [8]. However, when interpreting the data, the specific social, cultural, and environmental context must be taken into account. Indeed, translating and validating scales is an optimal way to obtain data that is comparable across countries, considering that the populations under study are similar. A robust comparison can suggest potential universal interventions that could be standardized. All things considered, the French version of the MHLS has not yet been developed.

### 1.2. Students’ Mental Health

Although university students report mental health conditions similar to their non-university peers [9], recent studies suggest an increase and severity of mental health problems in this population around the world in the last few years [10,11,12]. A systematic review has showed that students are particularly vulnerable to depression, stress, and anxiety problems [13]. Studying at the university represents a heavy stressor [14]; students face new challenges such as leaving their homes and being distant from their support networks, making independent decisions about their lives, and suffering academic pressure [15]. Among all academic disciplines, healthcare studies are particularly demanding. Extensive time and emotional commitment are necessary for students to devote to their training and this might cause distress [16]. Another risk factor is sex: being a female student increases the chances of having a mental health problem [17]. Environmental and genetic influences are at the origin of this difference [18].

The COVID-19 crisis has exacerbated students’ distress with containment measures increasing the prevalence of depressive and anxiety disorders [19]. As in other countries, French students have experienced and are still suffering from severe mental health problems due to the pandemic [20].

### 1.3. Students’ Mental Health Literacy: Data and Interventions

Improving students’ MHL may help them recognize signs of a mental health problem, seek for timely help, and fight against stigmatisation. Globally, untreated mental disorders are highly prevalent in students [11], and MHL is meant to enhance recovery. The few surveys using the MHLS have shown low levels of MHL in students. Two surveys have been conducted in the UK among medical students [21] and students from all disciplines [22]. In the first study, medical students reported a mean score of 127.69/160. In the second study, the mean MHLS score was slightly lower, 122.88/160. These studies used the complete 35-item MHLS as an already-validated tool in the English language. The mean MHLS score in Persian students was 69.59/106, using a 23-items MHLS version [23]. In Saudi Arabia, students scored 112.53/160 using the full MHLS [24]. In Malaysia, students’ mean score of the 35-item MHLS was 111.42/160. Overall, these data show a homogenous low level of MHL in students (around 40 points below the maximum score). No data exist on student’s MHL in France.

Research on MHL reported that males’ scores are lower than females’ [21], probably due to the prevailing gender role for males, i.e., the traditional masculine stereotype, rejecting any type of help and avoiding to disclose a personal mental health problem [25]. With the advancement of age, individuals tend to gain more knowledge and exposure to mental health issues, which might increase their level of MHL. In line with these results, students from late years of undergraduate programs report higher MHL scores [26]. The field of study also plays a pivotal role in MHL scoring. Studying psychology or medicine, for instance, is associated with high MHL scores since students of these disciplines have heard of and could define mental health problems based on their studies [27]. Previous research has shown that international students had a lower MHL score because of a different conceptualisation of MHL relating to cultural diversity [28]. Finally, low revenues correspond to limited MHL. This might be explained by a poor parental education level with negative attitudes toward the mentally ill [25].

In order to increase students’ MHL, several interventions have been implemented worldwide [29,30]. These included multi-channel communication (posters, social network posts, email, etc.) [31], digital videos [32], mental health first-aid programs [33], etc. There are several categories of MHL interventions: whole-of-community campaigns; community campaigns addressed exclusively to young people; university-based interventions teaching MHL; and programs training individuals in a mental health crisis.

To assess their effectiveness, some interventions underwent evaluation through different designs (ex. mixed-methods, randomized controlled trials, pre-post studies) using different outcome measures. The majority of the questionnaires employed for the evaluation were ad hoc ones [34], and validated scales were used only to a lesser extent [30]. The use of the MHLS to assess the improvement of MHL is recommended [5,35].

### 1.4. The Present Study

The objective of this study was to validate the French version of the MHLS, the MHSL-FR, in a group of French university students. The psychometric qualities of the translated and adapted scale were explored. The MHLS-FR is meant to be used to measure MHL in French students so as to inform the development of educational programs addressed to them. The scale should also be used to evaluate the effectiveness of these programs aimed to increase French students’ MHL.

## 2. Materials and Methods

### 2.1. Translation of the MHLS and Item Adaptation

The original MHLS was translated in French and back-translated in English following the standard methodologies for questionnaire translation [36]. Like the original scale, translated version initially contained 35 items. During a test phase, wording of the items was revised by 5 university students in order to make reading and comprehension accessible. The final version was read and approved by a panel of 6 public health researchers and mental health professionals.

### 2.2. Participants and Data Collection

Data collection took place between October and December 2019 at the University of Bordeaux, France. Self-administered questionnaires were distributed by peers to university students. A total of 550 participants were approached at the entrance of the university libraries and canteens. All participants had to be ≥18 years and French speaking. Before starting the questionnaire, respondents were informed about the content of the survey and asked for their consent. In total, 482 students out of 550 (87.6% response rate) completed the questionnaire (test). Their mean age was 20.46 (standard deviation, SD 2.46), and the majority were female (68.5%) and French students (89.4%). They were mostly healthcare students (37.6%) and attended the 1st year (33.2%). Their main source of income was family (63.7%) and they are for the most part living with less than 500 euros per month (46.9%). Among these students from the test phase, participants who completed the questionnaire twice (retest) were those who had voluntarily left their email address to be contacted again (*n* = 72). Finally, only 51 students out of 72 (70.8% response rate) agreed to participate in the retest with a final retention rate of 10.6% (51/482).

The study was conducted in compliance with the Declaration of Helsinki and with the approval of French Research Ethics Committee and the University of Bordeaux. Consent forms were obtained from the participants before data collection. The anonymity of the participants was maintained throughout the study with anonymized dataset.

### 2.3. Measurement and Missing Values

The full translated French version of the scale was identical to the original MHLS. Items were scored according to a Likert scoring system. The first dimension “ability to recognize specific disorders or different types of psychological distress” was composed of 8 items; the second dimension “knowledge and beliefs about risk factors and causes” of 2 items; the third dimension “knowledge and beliefs about self-help interventions” of 5 items; the fourth dimension “knowledge of where to seek information” of 4 items; the fifth dimension “wrong beliefs about mental health” of 9 items; and the sixth dimension “attitudes towards people with a mental health problem” of 7 items.

Items with a 4-point scale were rated 1—very unlikely/unhelpful, 4—very likely/helpful and for 5-point scale 1—strongly disagree/definitely unwilling, 5—strongly agree/definitely willing. The total score ranged from 35 to 160. Higher values corresponded to higher levels of MHL.

Only the item “A mental illness is not a real medical illness” (MAL_MED), due to an informatic error in the coding, presented 70.8% (341/482) missing values. We preferred reporting the descriptive and reliability analyses of this item with the 141 valid values by using pairwise case exclusion when introduced in the factorial models.

### 2.4. Data Analyses

We followed the procedure suggested by the Consensus-based Standards for the Selection of Health Measurement Instruments (COSMIN) [37,38].

First, we evaluated the facet validity of the French version of the MHLS through descriptive statistics and the judgement of a panel of experts (professional psychologists, epidemiologists, health professionals, researchers, and lecturers). An online 8-item checklist was sent to the experts to rate the MHLS-FR according to the criteria of acceptability, practicality, and relevance.

Second, we analysed the distribution of the items per dimension through percentages. For sake of clarity, we use the term “factor” when referring to our scale and “dimension” when referring to the original MHLS. The two terms are actually synonyms and explain to the same concept. Response options were reported to assess ceiling effect. We used descriptive methods to evaluate the relevance and the comprehensiveness of the items in the population under study. We used sex and field of study as the reference variables to distinguish the 35 items. Sex was used as a well-known variable influencing mental health status and, consequently, also familiarity with mental health topics. Field of study was used as a variable that characterizes students; we wanted to observe differences between students with high knowledge of health and psychology subjects versus students from other disciplines. We recoded the items so that values from 0 to 2 or from 0 to 3 were considered as low MHL (0) and >2 or >3 as higher MHL. When present, the answer “I don’t know” was scored as 0.

Third, we performed the test-retest reliability using the intraclass correlation coefficient (ICC) [39] calculated in the sub-sample of 51 students of the retest. Values < 0.40, 0.41–0.75, and >0.75 were indicative of bad, good, and very good reliability, respectively [39]. Calculations were made for each item and for each factor before and after the validation process.

Fourth, we assessed dimensionality and structural validity through Exploratory Structural Equation Model (ESEM) [40] in order to allow items to present cross-loadings [41] between obtained factors so as to provide a better fit compared to the Confirmatory Factor Analysis (CFA). We aimed to identify the dimensions proposed by O’Connor and Casey [5] so as to confirm them by using CFA models. In both ESEM and CFA models we used the polychoric correlation matrix [42] between the items of the scale and the Weighted Least-Square Mean and Variance (WLSMV) method. For evaluating both models, we used two types of criteria: related to the items and related to the global model. For acceptable items, we used the factor loadings superior to 0.3 and the communalities (R-square) superior to 0.25. For the global model, we used the standard criteria: Chi-square test (χ^2^); the Comparative Fit Index (CFI), and Tucker–Lewis Index (TLI) with values >0.90 considered adequate (preferably >0.95); Root Mean Square Error of Approximation (RMSEA) with cut-off values of <0.8 (preferably <0.5); and Standardized Root Mean Square Residual (SRMR) where values <0.10 were considered as acceptable. The correlations between factors were evaluated using Pearson’s r.

Fifth, concerning the structural validity, for the analysis of the Measurement Invariance (MI) of the obtained factors [43] across sex groups, we used CFA multigroups models for the ordinal categorical variables [44], by using a top-down approach [45] to compare the two added models [46]. We started with the most restrictive or complete invariance model where all factor loadings and thresholds remain invariant within groups (H0 or scale invariance). Than we further compared results with the model where we released the equality restrictions of the previous parameters in models with the same factorial structure for each group (H1 or configural invariance). In order to evaluate the MI, we used the change criterion of the χ^2^ [47] together with other alternative adjustment indicators [48,49] that suggested to accept MI for changes minor to 0.01 in CFI, minor to 0.015 in RMSEA, and minor to 0.030 in SRMR.

Sixth, for the reliability analysis and the internal consistency of the scale and the factors, we used the Cronbach’s alpha coefficient [50] and the McDonald’s omega [51]. Well-accepted values for both coefficients are between 0.70 and 0.90, respectively [52]. We also calculated the standard error of measurement (SEM) via the (SD) [53].

Finally, we used hypothesis tests using Student’s *t*-test or ANOVA and Pearson’s correlation coefficients to assess the convergent and discriminant validity of the total scale score and of the obtained factors with the sociodemographic variables sex, age, year, and field of study, being an international student, main source of income, and monthly all-inclusive resources. All *p* values were two-tailed, and we considered a *p* < 0.05 to be statistically significant. Analyses were conducted using SPSS v24 (SPSS, Chicago, IL, USA) and MPlus v8.4.

## 3. Results

### 3.1. Face Validity

Experts rated very high acceptability at 48.9%, very high practicality at 68.9%, and very high relevance at 46.6%. Remaining percentages corresponded to high or medium values for each criterion.

### 3.2. Distribution of the Items

We analysed the distribution of the items and the psychometric proprieties of the original MHLS with the 35 translated items. Acronyms and short definitions are available in Table S1 in the Supplementary Material. Mean score was 90.52 (SD = 8.95, Minimum = 60.00, Maximum = 115.00, 95% CI = 89.72–91.72).

Table S2 in the Supplementary Material shows that all the answer options of the items were used (e.g., from “very unlikely” to “very likely”). This suggests that some items were more difficult and had lower capacity of discrimination.

We analysed the differences across the answers according to sex and field of study chosen a priori among covariates (Table S3 in the Supplementary Material). We observed that PHO_SOC, DYS, BIPOL, TCC, MAL_MED, PAS_FORT, and POLI_MAL were significantly different (*p* values from 0.002 to 0.036) between females and males. PHO_SOC, ANX_GEN, DYS, FEM_RIS_MAL, TCC, DIF_ANX_R, DANGER_R, TRAIT_PAS_EFF_R, and MARIA_MAL were significantly different across fields of study (*p* values from 0.001 to 0.047). Overall, the majority of the 35 items (between 75 to 80%) were not different across these two sociodemographic variables, i.e., items were independent.

### 3.3. Test Retest

Table S2 in the Supplementary Material shows the ICCs for each item. The majority of the items presented acceptable values. There was about 63% of items with good ICCs and 37% of bad items, although only three items had ICCs below 0.2: TROU_PERSO, TCC, and DIF_ANX.

### 3.4. Dimensionality

We used ESEM models from six factors, and we obtained models whose factors had factor loadings superior to 0.3 and items with communalities (R-square) superior to 0.25. For this, we eliminated items or factors so as to obtain good fit indicators, until we obtained the possible dimension or sub-dimensions of the original MHLS in our study population.

Table 1 shows the different models we obtained until the proposed criteria were met. Within them, we had to eliminate the dimension of “knowledge and beliefs about self-help interventions”, explained by the items SOMM and DIF_ANX, in addition to other 5 items that did not reach a communality higher than 0.25.

Table 2 presents the ESEM model 5 whose RMSEA, CFI, TLI, and SMRM fit indicators were excellent. Through the ESEM, we meant to explore whether the factors found in our study corresponded to the dimensions of the original MHLS.

In model 5, the three dimensions obtained by O’Connor and Casey on “knowledge of risk factors and causes”, “knowledge and beliefs about self-help intervention”, and “knowledge of where to seek information” were present in our population, and sensibly with the same items than the original scale (except for the item CONFI_PROB_ENT). Regarding the other two dimensions, the factor “ability to recognize disorders” was divided into two sub-factors that appeared highly significantly correlated (r = 0.401), and the factor “stigmatisation” was divided into four sub-factors, two related to “wrong beliefs about mental health” and the other two related to “attitudes towards people with a mental health problem”. It is worth noting that the correlation (r = 0.407) between the two sub-factors corresponding to “stigmatisation” was significant. Factor 2 included one sub-factor of the “ability to recognize disorders” and of “knowledge and beliefs about self-help interventions”. Table 2 presents the factors identified in our study crossed with the dimensions of MHLS. Factors with high correlations (ex. F1 and F2) allows to think that they both define the same dimension corresponding to the MHLS related dimension. Correlations between factors inform the following CFA.

### 3.5. Structural Validity

First, dimensional analyses were performed through the ESEM models. Then, we used different CFA models in order to confirm the factors or sub-factors obtained by the original MHLS. In the search for the best CFA model, we continued to use the criteria described for factor loadings and communalities, and eliminated the items FRAG_PERS_21 and MAL_MED_22 for having low communalities. Table 3 shows the model with the 26 items and the six factors, where there were still three items (PHO_SOC_1, ANX_GEN_2, and HOM_RIS_ANX_10) with a communality inferior to 0.25 but with a global acceptable fit (RMSEA = 0.054, CFI = 0.909, TLI = 0.895 and SMRM = 0.061) and a sound interpretation of the obtained factors (i.e., coherence of the items and comparability with the dimensions of the original scale). The Table crosses again the factors we obtained with the dimensions of the original scale. Significant correlations confirmed the division of the original dimensions.

The factors obtained in Table 4 (visual representation in Figure S1) show the presence of 5 of the 6 dimensions obtained by O’Connor and Casey, and a strong correlation between them except for dimension 2 “knowledge of risk factors and causes”.

The fit coefficients of the parameters of the simple and multigroup models were acceptable, and the changes in the absolute values of the fit indices were within the range proposed by Chen [49] for accepting the MI. The description of the distribution of each of the dimension scores obtained and of the total scale as well as the indicators of reliability, internal consistency and test-retest is shown in Table S4. Standard error of measurement was also calculated and found to be 3.03.

### 3.6. Internal Consistency

Values of the internal consistency of the completed questionnaire and of the six dimensions of the original MHLS scale are reported in Table 5. Only the complete questionnaire and factor 7 reported a good internal consistency. Nevertheless, we obtained factors with alpha values that were low or negative (due to reversed items in the translated scale) ranging from −0.827 to 0.815.

In order to analyse the effect of the percentage of missing values for the item MAL_MED, we also obtained the alpha coefficient of the factor 6 of the complete questionnaire without including MAL_MED (i.e., with *n* = 482), obtaining a similar result: 0.583 for the factor 6 and 0.765 in total.

### 3.7. Convergent and Discriminant Validity

We tested the hypotheses that we could obtain different scores across sociodemographic characteristics. We hypothesized that female students presented higher scores, however this hypothesis was not confirmed (*p* = 0.212). For age, we hypothesized that older students presented a higher MHL score. Correlation coefficients were small even if some of them were significant maybe because of the sample size, thus confirming our hypothesis. International students presented overall lower scores of MHL than national students (*p* = 0.018). Concerning the field of study, the maximum score was reported in students from Human and Social Sciences, including Psychological Studies (93/119). The second maximum score was reported in students from Health Studies (91.2/119). No statistically significant differences were found with regards to the variables main source of income and monthly all-inclusive resources, *p* = 0.117 for both variables.

All this considered, the MHLS-FR was composed of 26 items with a score from 0 to 119. The items were: PHO_SOC_1; ANX_GEN_2; TROU_PERSO_4; DYS_5; AGORA_6; BIPOL_7; DRUG_8; FEM_RIS_MAL_9; HOM_RIS_ANX_10; TCC_13; CONFI_DANG_IMM_14; INFO_MAL_16; ORDI_INFO_17; QUES_MAL_18; RESS_INFO_19; FREQ_DEV_24R; PAS_FORT_26R; NO_AID_27R; TRAIT_PAS_EFF_28R; HAB_MAL_29; DISC_MAL_30; AMI_MAL_31; TRAV_MAL_32; MARIA_MAL_33; POLI_MAL_34; and EMB_MAL_35. Six factors were represented: “ability to recognize disorders” (7 items), “knowledge of sex as a risk factor” (2 items), “knowledge and beliefs about self-help interventions” (2 items), “knowledge of where to seek information” (4 items), and “stigmatisation” (11 items). The acronyms and grouped factors are explained in Table S1 in the Supplementary Material.

## 4. Discussion

The French version of the MHLS presented good psychometric proprieties and was capable of measuring comprehensively MHL in the target population of students. The MHLS-FR was composed of 26 items, 9 less than in the original MHLS. The reduction of the items can be explained by the fact that some of them were highly correlated and could be associated one with the other given their similar contents (e.g., “mental health is fragility” and “mental health is an illness”). In our study, some items were not discriminatory of a specific sub-factor and were removed based on both statistical features and interpretability of the results. The six factors demonstrated reliable psychometric proprieties and fully covered the original representation of the MHL construct. By means of comparison, the Pakistani version of the MHLS was composed of 34 items [54], the Iranian one of 29 items [55], and the Arabic one of 28 items [56]. In the studies conducted in China [57], Portugal [58], South Africa, and Zambia [59], all items were kept.

Concerning descriptive statistics, the mean MHL score calculated in the sample was 90.52/119. This score was higher than in previous studies on students’ MHL [21,22,23,24] and even in the study of O’Connor and Casey [5] where the mean score was 127.38/160. Variables such as countries where the study was conducted, cultures, educational training, and language might have influenced the results. Translation of the items may have had an impact on the interpretability of the items. The difficulty of the items must also be considered: the complete scale might include more complex items for young adults compared to the 26-item MHLS-FR. Furthermore, the sample of this study was unbalanced: there were higher proportions of female students and of healthcare students. As reported in the literature [21,27], the MHL scores of these populations are higher than their peers. This study is not representative of all French students and a larger survey would better capture their MHL scores. In any case, a score 30 points lower than the maximum score is not satisfactory and, there is room for improvement. Increasing MHL scores remains a global public health priority [60]. Interventions are needed among students even if at different degrees depending on the MHL scores of concerned populations.

In this study, the Cronbach’s alpha was acceptable, 0.744. It scored better in our final scale with 26 items compared to the original scale (0.765). This corroborates our findings: the new MHLS-FR tool was slightly more informative among French students than the original full scale. By means of comparison, the Cronbach’s alpha level of the original scale by O’Connor and Casey [5] was of 0.873. For test-retest reliability, results were positive: r(51) = 0.869, *p* < 0.001. The standard error of measurement (SEM) was 4.03. In the original scale [5], the retest results also showed good reliability (r(69) = 0.797, *p* < 0.001). To the same extent as the scores calculated in other versions of the scale, these results must be interpreted with caution. The scales are not fully comparable: translation and different interpretation of the items might have influenced these results.

For convergent and divergent validity, scores of MHL were different only for the variables age and nationality, thus showing that the scale measured MHL independently from sociodemographic characteristics. Similar results were found in the Pakistani study [54].

Strengths of this study include the fact that it produced a golden standard for measuring MHL in a French-speaking population. The use of this scale will benefit several communities of French-speaking young people given the increasing importance of MHL in the field of mental health promotion and disease prevention. The MHLS-FR can be used as a solid and reliable tool to measure the effectiveness of interventions aimed at improving MHL of young people. Its items and factors can also inform the design and development of such interventions. Contents to be covered should be those where students have the lower scores, the objective being to empower them for better mental health outcomes through a process-based learning approach. For instance, increased knowledge makes the student confident to talk to someone about depression, which leads to help-seeking behaviour. To the same extent knowledge leads to self-efficacy, which, in turn, leads to healthy behaviours. The study also answers the need to extend the generalisability of the Australian MHLS with other international samples and provides hints for developing statistically robust norms that can be used to guide the use of this scale [5].

This study is not without limitations. The sample of the retest was recruited on a voluntary basis and was not comparable to the larger sample of the first phase. This limited number of individuals for the retest phase can be explained by the difficulties researchers often face for retaining young people in repeated measure surveys [61,62]. However, the number of 51 was enough for the retest based on statistical power [63]. One value was missing for several respondents, but it was finally removed from the factorial analysis, thus not impacting on the final results. In the final scale of six factors, three items presented (R-square) inferior to 0.25, but, for the sake of interpretability, we included them in the tool.

## 5. Conclusions

The main significance of the study is to provide the first French instrument to measure MHL in students. Its good psychometric proprieties guarantee that the MHLS-FR can be used to collect reliable and robust data. With only 26 items, it is easy and quick to use. For this, it might be included in large surveys on students’ health to produce information advocating for the design and development of MHL education programs. Student Health Services will be alerted to the need for implementing these programs. More broadly, the University Executive Boards would be sensitised to students’ MHL.

The MHLS-FR is also meant to be used to evaluate the impact of MHL education programs. It should be administered to assess the change in MHL score before and after the interventions following different designs: randomised controlled trial, pre-post-design, longitudinal study, etc. Effective interventions would promote positive attitudes towards mental health, facilitate access to care, promote help-seeking behaviour and reduce stigma.

Finally, further research is needed to replicate the present findings in other populations. The MHLS-FR offers considerable benefits in mental health research and practice, and its use should be extended beyond students.

## Figures and Tables

**Table 1:** ESEM models and indicators.

		Chi-Squ. Base	Chi-Squ	df	RMSEA	90 CI	CFI	TLI	SMRM
MODEL 1	ESEM 35 items; 6 factors	5,339,633	630,037	400	0.035	0.029 0.040	0.952	0.928	0.041
MODEL 2	ESEM 35 items; 7 factors	5,339,633	554,691	371	0.032	0.026 0.037	0.961	0.938	0.038
MODEL 3	ESEM 35 items; 8 factors	5,339,633	490,922	343	0.03	0.024 0.036	0.969	0.946	0.033
MODEL 4	ESEM 32 items; 8 factors Elim: DEPRESS, DIF_ANX, DANGER	5,205,246	371,601	343	0.028	0.021 0.035	0.978	0.959	0.031
MODEL 5	ESEM 28 items; 8 factors Elim: CONFI_PROB_ENT, SOMM, VOU_SOR, DIR_PER	4,850,961	233,366	297	0.024	0.014 0.033	0.989	0.976	0.026

**Table 2:** MHLS-FR dimensions based on model 5.

	Factors of the MHLS-FR								R Square	O’Connor and Casey’s MHLS Dimensions
	F1	F2	F3	F4	F5	F6	F7	F8		
PHO_SOC_1	**0.625**	0.031	0.117	−0.006	0.012	−0.007	−0.020	−0.022	0.451	Ability to recognize disorders
ANX_GEN_2	**0.575**	0.094	−0.007	0.046	−0.028	0.007	−0.049	−0.009	0.394	
TROU_PERSO_4	0.083	**0.407**	0.126	0.165	−0.073	0.021	−0.050	−0.045	0.309	
DYS_5	0.005	**0.538**	−0.024	0.166	−0.040	−0.203	0.047	−0.066	0.295	
AGORA_6	0.083	**0.635**	−0.047	−0.103	0.024	0.039	0.040	−0.001	0.449	
BIPOL_7	0.091	**0.447**	0.041	0.017	0.039	0.052	−0.011	−0.123	0.281	
DRUG_8	0.018	**0.660**	0.020	0.001	0.088	0.010	0.002	0.057	0.474	
FEM_RIS_MAL_9	−0.040	0.224	−0.011	0.024	**−0.562**	−0.017	−0.029	−0.022	0.368	Knowledge of risk factors and causes
HOM_RIS_ANX_10R	−0.066	0.101	−0.030	0.036	**0.645**	0.018	−0.005	−0.039	0.433	
TCC_13	0.073	**0.653**	0.003	0.022	−0.008	−0.272	0.058	0.049	0.429	Knowledge and beliefs about self-help interventions
CONFI_DANG_IMM_14	−0.169	**0.516**	0.120	−0.019	−0.018	0.169	−0.093	0.135	0.377	
INFO_MAL_16	−0.091	0.097	**0.763**	−0.066	0.025	−0.069	0.000	−0.021	0.581	Knowledge of where to seek information
ORDI_INFO_17	0.012	0.000	**0.683**	−0.015	0.025	0.053	−0.025	0.110	0.504	
QUES_MAL_18	0.080	−0.138	**0.377**	0.065	−0.020	−0.065	0.398	0.041	0.373	
RESS_INFO_19	0.036	0.000	**0.648**	0.069	−0.036	0.154	0.086	−0.051	0.516	
FRAG_PERS_21R	−0.018	−0.005	0.030	0.106	0.027	**0.460**	0.040	−0.040	0.251	Stigmatisation
MAL_MED_22R	0.491	0.043	0.001	−0.197	0.001	**0.395**	0.035	0.070	0.413	
FREQ_DEV_24R	0.000	−0.019	−0.005	0.298	0.068	**0.566**	−0.012	0.036	0.498	
PAS_FORT_26R	0.097	0.020	−0.024	0.118	−0.021	**0.523**	0.436	−0.043	0.597	
NO_AID_27R	−0.076	0.008	0.027	−0.051	0.056	0.023	**0.920**	0.016	0.865	
TRAIT_PAS_EFF_28R	−0.045	0.057	0.009	−0.033	−0.017	0.357	**0.390**	0.013	0.336	
HAB_MAL_29	−0.034	0.052	0.013	**0.532**	0.083	0.079	−0.010	0.134	0.410	
DISC_MAL_30	−0.031	0.164	−0.016	**0.806**	0.029	−0.008	0.031	−0.010	0.705	
AMI_MAL_31	0.011	0.021	0.006	**0.821**	−0.051	0.025	−0.022	0.085	0.752	
TRAV_MAL_32	0.027	−0.040	0.009	**0.608**	−0.019	0.019	0.039	0.313	0.639	
MARIA_MAL_33	0.128	−0.059	0.037	0.288	0.195	−0.071	−0.011	**0.526**	0.561	
POLI_MAL_34	−0.017	0.074	−0.010	−0.008	0.003	0.035	−0.013	**0.806**	0.661	
EMB_MAL_35	−0.028	0.025	−0.034	0.112	−0.047	0.012	0.063	**0.691**	0.561	
	Correlations between factors									
F1	1.000									
F2	0.401 *	1.000								
F3	0.235 *	0.267 *	1.000							
F4	0.192 *	0.164 *	0.145 *	1.000						
F5	−0.073	−0.022	0.013	0.050	1.000					
F6	0.010	0.372 *	0.087	0.196	0.184	1.000				
F7	0.084	0.105	0.169 *	0.154 *	0.106	0.158 *	1.000			
F8	0.044	0.060	0.126 *	0.407 *	0.102	0.141	0.123 *	1.000		

* significant (*p* < 0.05). Factor 1: Ability to recognize disorders. Factor 2: Ability to recognize disorders + Knowledge and beliefs about self-help interventions. Factor 3: Knowledge of where to seek information. Factor 4: Stigmatisation. Factor 5: Knowledge of risk factors and causes. Factor 6–8: Stigmatisation. Values in bold correspond to factor loadings >0.3 which are necessary to group items in a factor.

**Table 3:** Final MHLS-FR version with 26 items and 6 factors.

	Factors of the MHLS-FR						R Square	O’Connor and Casey’s MHLS Dimensions
	F1	F2	F3	F4	F5	F6		
PHO_SOC_1	0.434						0.188	Ability to recognize disorders
ANX_GEN_2	0.427						0.183	
TROU_PERSO_4	0.589						0.347	
DYS_5	0.486						0.236	
AGORA_6	0.585						0.342	
BIPOL_7	0.490						0.240	
DRUG_8	0.681						0.464	
FEM_RIS_MAL_9		0.960					0.922	Knowledge of risk factors and causes
HOM_RIS_ANX_10		0.368					0.136	
TCC_13			0.560				0.314	Knowledge and beliefs about self-help interventions
CONFI_DANG_IMM_14			0.514				0.264	
INFO_MAL_16				0.615			0.378	Knowledge of where to seek information
ORDI_INFO_17				0.697			0.485	
QUES_MAL_18				0.507			0.257	
RESS_INFO_19				0.756			0.571	
FREQ_DEV_24R					0.653		0.427	Stigmatisation
PAS_FORT_26R					0.775		0.601	
NO_AID_27R					0.560		0.314	
TRAIT_PAS_EFF_28R					0.526		0.277	
HAB_MAL_29						0.623	0.388	
DISC_MAL_30						0.790	0.625	
AMI_MAL_31						0.819	0.670	
TRAV_MAL_32						0.783	0.612	
MARIA_MAL_33						0.655	0.430	
POLI_MAL_34						0.618	0.382	
EMB_MAL_35						0.620	0.384	
	Correlations between factors							
F1	1.000							
F2	0.177 *	1.000						
F3	0.993 *	0.187	1.000					
F4	0.383 *	0.004	0.428 *	1.000				
F5	0.319 *	−0.132	0.306 *	0.378 *	1.000			
F6	0.257 *	−0.062	0.321 *	0.252 *	0.413 *	1.000		

* significant (*p* < 0.05). F1: Ability to recognize disorders. F2: Knowledge of risk factors and causes. F3: Knowledge and beliefs about self-help interventions. F4: Knowledge of where to seek information. F5 and F6: Stigmatisation.

**Table 4:** Measurement of invariance in the CFA model.

	WLSMV χ^2^ (df)	CFI	RMSEA	SRMR	χ^2^ (df)	*p*	CFI	RMSEA	SRMR
Baseline Models									
Males (*n* = 152)	462,441 (284)	0.852	0.064	0.09					
Females (*n* = 330)	526,535 (289)	0.922	0.05	0.066					
Measurement Invariance for sex									
Scalar model (H0)	1,063,427 (653)	0.898	0.051	0.078					
Configural model (H1)	1,002,902 (580)	0.895	0.055	0.076	92.519 (73)	0.0612	−0.003	0.004	−0.002

H0: factor loadings and thresholds free across groups, scale factors fixed at one in all groups, and factor means fixed at zero in all groups. H1: factor loadings and thresholds constrained to be equal across groups, scale factors fixed at one in one group, and free in the other groups, and factor means fixed at zero in one group and free in the other groups.

**Table 5:** Scores of the scale and the dimensions: description, reliability, internal consistency, and test-retest reliability.

	Total Mean (SD)	Range	Cronbach’s Alpha	McDonald Omega	ICC: IC95% (*n* = 51)
F1	22.08 (2.78)	10–28	0.643	0.872	0.772 IC95% = (0.601; 0.870)
F2	4.56 (1.33)	2–8	0.453	0.625	0.637 IC95% = (0.365; 0.793)
F3	6.35 (1.10)	2–8	0.343	0.666	0.291 IC95% = (−0.242; 0.595)
F4	15.05 (2.77)	5–20	0.608	0.797	0.908 IC95% = (0.838; 0.947)
F5	17.12 (2.40)	8–20	0.595	0.796	0.793 IC95% = (0.637; 0.882)
F6	25.36 (5.15)	11–35	0.815	0.873	0.867 IC95% = (0.7671; 0.924)
Total Scale	90.52 (8.95)	60–115	0.765	0.961	0.869 IC95% = (0.770; 0.925)

## Data Availability

The data that support the findings of this study are available from the corresponding author, I.M., upon reasonable request.

## Supplementary Materials

The following supporting information can be downloaded at: https://www.mdpi.com/article/10.3390/bs12080259/s1, Figure S1: Representation of the confirmatory factor analyses. Table S1:List of acronyms inspired by the original MHLS according to the classification of O’Connor and Casey (2015). Table S2: Distribution (%) of the answers of the items of the proposed scale (with no inversed items) and test-retest reliability. Table S3: Distribution (%) of the answers of the items of the proposed scale MHLS (with inversed items). Table S4: Scores of the scale and dimensions: description, reliability, internal consistency and test-retest reliability.
