# Supplementary material for: Validation of the Mental Health Literacy Scale in French University Students
Source: Behav Sci (Basel). 2022 Jul 28;12(8):259. doi: 10.3390/bs12080259 (PMC9404754; doi:10.3390/bs12080259)
Supplement: Supplementary file 1 [file behavsci-12-00259-s001.zip › behavsci-1820560-supplementary.pdf]

SUPPLEMENTARY MATERIAL

Table S1. List of acronyms inspired by the original MHLS according to the classification of O'Connor and Casey (2015)

	Acronym	Meaning	Dimension
1	PHO_SOC	Definition of social phobia	Ability to recognise specific disorders or different types of psychological distress
2	ANX_GEN	Definition of general anxiety	
3	DEPRESS	Definition of severe Depression	
4	TROU_PERSO	Definition of personality troubles	
5	DYS	Definition of dysthymia	
6	AGORA	Definition of agoraphobia	
7	BIPOL	Definition of bipolar disorder	
8	DRUG	Definition of drug addiction	
9	FEM_RIS_MAL	Females are more at risk	Knowledge and beliefs about risk factors and causes
10	HOM_RIS_MAL	Males are more at risk	
11	TCC	Cognitive-behavioural therapy	Knowledge and beliefs about self-help interventions
12	CONFI_DANG_IMM	Confidentiality about immediate danger	
13	CONFI_PROB_ENT	Confidentiality about no danger	
14	SOMM	Sleep quality	
15	DIF_ANX	Avoid anxiety	
16	INFO_MAL	Access to health information	Knowledge of where to seek information
17	ORDI_INFO	Digital access to health information	
18	QUES_MAL	Easiness to ask information	
19	RESS_INFO	Confidence in information sources	
20	VOUL_SORT	Mental health can be self-cured	Wrong beliefs about mental health
21	FRAG_PERS	Mental health is fragility	
22	MAL_MED	Mental health is an illness	
23	DANGER	Impaired mental health is dangerous	
24	FREQ_DEV	Avoid contacting people with mental health	
25	DIR_PERS	Not disclosing a mental illness	
26	PAS_FORT	Not being strong with a mental illness	
27	NO_AID	Not asking for help with a mental illness	
28	TRAIT_PAS_EFF	Treatment is useless	
29	HAB_MAL	Living with someone with a mental illness	Attitudes towards people with a mental health problem
30	DISC_MAL	Discussing with someone with a mental illness	
31	AMI_MAL	Friendship with someone with a mental illness	
32	TRAV_MAL	Working with someone with a mental illness	
33	MARIA_MAL	Marrying someone with a mental illness	
34	POLI_MAL	Voting for someone with a mental illness	
35	EMB_MAL	Employing someone with a mental illness	

Table S2. Distribution (%) of the answers of the items of the proposed scale (with no inversed items) and test-retest reliability

	Original MHLS (n=482)					Test-retest reliability CI (CI95%)
	**Very unlikely**	**Unlikely**	**Likely**	**Very Likely**		
**Ability to recognise specific disorders or different types of psychological distress ***						
**PHO_SOC**	6.0	24.1	61.8	8.1		0.511 CI95%= (0.276; 0.688)
**ANX_GEN**	1.9	12.9	67.0	18.3		0.404 CI95%= (0.147; 0.610)
**DEPRESS**	3.7	25.5	54.6	16.2		0.251 CI95%= (-0.02; 0.490)
**TROU_PERSO**	4.4	7.1	43.8	44.8		-0.105 CI95%= (-0.36; 0.173)
**DYS**	3.3	13.3	70.1	13.3		0.414 CI95%= (0.158; 0.617)
**AGORA**	2.7	7.9	42.9	46.5		0.504 CI95%= (0.268; 0.683)
**BIPOL**	3.9	10.8	49.0	36.3		0.270 CI95%= (-0.00; 0.505)
**DRUG**	2.3	3.3	25.7	68.7		0.240 CI95%= (-0.03; 0.481)
**Knowledge and beliefs about self-help interventions ***						
**FEM_RIS_MAL**	16.6	32.8	37.8	12.9		0.626 CI95%= (0.426; 0.768)
**HOM_RIS_MAL**	20.1	53.3	24.3	2.3		0.555 CI95%= (0.332; 0.719)
**Knowledge and beliefs about self-help interventions***						
	**Very unhelpful**	**Unhelpful**	**Helpful**	**Very helpful**		
**TCC**	2.1	12.7	71.0	14.3		0.179 CI95%= (-0.09; 0.430)
**CONFI_DANG_IMM**	5.0	5.4	36.5	53.1		0.296 CI95%= (0.025; 0.526)
**CONFI_PROB_ENT**	49.4	27.8	15.6	7.3		0.238 CI95%= (-0.03; 0.479)
**Knowledge of self-treatment***						
**SOMM**	1.0	2.3	39.6	57.1		0.281 CI95%= (0.008; 0.514)
**DIF_ANX**	9.5	36.3	40.0	14.1		0.172 CI95%= (-0.10; 0.424)
**Knowledge of where to seek information ****						
	**Strongly Disagree**	**Disagree**	**Neither agree or disagree**	**Agree**	**Strongly agree**	
**INFO_MAL**	2.7	23.2	19.3	42.9	11.8	0.550 CI95%= (0.325; 0.715)
**ORDI_INFO**	2.1	10.6	6.8	52.1	28.4	0.574 CI95%= (0.356; 0.732)
**QUES_MAL**	3.9	20.3	10.6	38.6	26.6	0.755 CI95%= (0.606; 0.852)
**RESS_INFO**	1.5	5.2	6.4	56.4	30.5	0.438 CI95%= (0.186; 0.634)
**Attitudes towards people with a mental health problem ****						
**VOUL_SORT**	22.4	42.7	18.3	12.7	3.9	0.565 CI95%= (0.344; 0.726)
**FRAG_PERS**	29.0	31.1	12.0	25.1	2.7	0.431 CI95%= (0.178; 0.630)
**MAL_MED (n=141)**	67.4	27.0	2.1	2.1	1.4	0.280 CI95%= (0.007; 0.514)
**DANGER**	2.9	6.6	10.6	55.4	24.5	0.223 CI95%= (-0.05; 0.467)

**FREQ_DEV**	69.3	24.5	4.1	1.9	0.2	0.646 CI95%= (0.452; 0.781)
**DIR_PERS**	22.2	42.3	17.4	16.2	1.9	0.534 CI95%= (0.305; 0.704)
**PAS_FORT**	62.2	25.3	3.3	8.3	0.8	0.530 CI95%= (0.299; 0.701)
**NO_AID**	44.8	35.7	9.5	8.9	1.0	0.283 CI95%= (0.010; 0.516)
**TRAIT_PAS_EFF**	30.7	45.0	16.4	6.8	1.0	0.590 CI95%= (0.376; 0.743)
**Stigmatisation****						
	**Definitely unwilling**	**Probably unwilling**	**Neither unwilling or willing**	**Probably willing**	**Definitely willing**	
**HAB_MAL**	9.1	20.1	22.2	32.0	16.6	0.489 CI95%= (0.249; 0.672)
**DISC_MAL**	0.8	6.0	7.1	45.0	41.1	0.387 CI95%= (0.127; 0.597)
**AMI_MAL**	1.5	7.9	14.1	47.3	29.3	0.615 CI95%= (0.410; 0.760)
**TRAV_MAL**	1.5	12.9	18.7	45.6	21.4	0.544 CI95%= (0.317; 0.711)
**MARIA_MAL**	5.6	12.2	19.5	34.9	27.8	0.584 CI95%= (0.369; 0.738)
**POLI_MAL**	14.3	21.6	24.9	26.3	12.9	0.748 CI95%= (0.596; 0.847)
**EMB_MAL**	2.5	15.1	23.4	44.4	14.5	0.664 CI95%= (0.477; 0.793)

*Score from 1 to 4
**Score from 1 to 5

Table S3. Distribution (%) of the answers of the items of the proposed scale MHLS (with inversed items)

	Total	Male	Female	p value	Hum.SS	TecSc	HelathS	Law-E	p value
**Ability to recognize disorders: Likey or Very Likely (≥3)**									
**PHO_SOC**	69.9%	63.8%	72.7%	**0.047**	82.5%	60.0%	63.3%	75.9%	**0.002**
**ANX_GEN**	85.3%	81.6%	87.0%	**0.121**	91.2%	85.3%	78.4%	88.5%	**0.039**
**DEPRESS**	70.7%	75.0%	68.8%	**0.164**	68.4%	75.8%	69.1%	70.2%	**0.673**
**TROU_PERS**	88.6%	86.8%	89.4%	**0.413**	91.2%	87.4%	87.1%	89.5%	**0.795**
**DYS**	83.4%	75.7%	87.0%	**0.002**	84.2%	77.9%	79.1%	89.0%	**0.041**
**AGORA**	89.4%	87.5%	90.3%	**0.353**	91.2%	88.4%	87.8%	90.6%	**0.809**
**BIPOL**	85.3%	80.3%	87.6%	**0.035**	87.7%	81.1%	82.0%	89.0%	**0.178**
**DRUG**	94.4%	92.8%	95.2%	**0.289**	96.5%	90.5%	92.8%	96.9%	**0.111**
**Knowledge of risk factors and causes: Likey or Very Likely (≥3)**									
**FEM_RIS_MAL**	50.6%	44.1%	53.6%	**0.051**	47.4%	46.3%	43.9%	58.6%	**0.039**
**HOM_RIS_MAL_R***	73.4%	69.7%	75.2%	**0.211**	68.4%	76.8%	70.5%	75.4%	**0.515**
**Knowledge of professional help available: Likey or Very Likely (≥3)**									
**TCC**	85.3%	78.9%	88.2%	**0.008**	84.2%	75.8%	82.0%	92.7%	**0.001**
**CONFI_DANG_IMM**	89.6%	88.8%	90.0%	**0.692**	93.0%	90.5%	86.3%	90.6%	**0.461**
**CONFI_PROB_ENT_R***	77.2%	73.7%	78.8%	**0.215**	84.2%	74.7%	74.8%	78.0%	**0.487**
**Knowledge of self-treatment Agree or Strongly agree (≥3)**									
**SOMM**	96.7%	96.1%	97.0%	**0.602**	96.5%	96.8%	94.2%	98.4%	**0.221**
**DIF_ANX _R***	45.9%	39.5%	48.8%	**0.057**	56.1%	52.6%	36.0%	46.6%	**0.021**
**Knowledge of where to seek information**									
**INFO_MAL**	54.8%	59.2%	52.7%	**0.184**	59.6%	60.0%	46.0%	57.1%	**0.099**
**ORDI_INFO**	80.5%	84.9%	78.5%	**0.100**	86.0%	86.3%	77.7%	78.0%	**0.205**
**QUES_MAL**	65.1%	61.8%	66.7%	**0.302**	66.7%	67.4%	59.7%	67.5%	**0.466**
**RESS_INFO**	86.9%	85.5%	87.6%	**0.535**	87.7%	88.4%	82.7%	89.0%	**0.377**
**Attitudes towards people with a mental health problem: Agree or Strongly agree (≥4)**									
**VOUL_SORT_R***	65.1%	65.8%	64.8%	**0.840**	63.2%	62.1%	60.4%	70.7%	**0.219**
**FRAG_PERS_R***	60.2%	55.9%	62.1%	**0.196**	54.4%	62.1%	58.3%	62.3%	**0.679**
**MAL_MED_R* (n=141)**	94.3%	84.4%	97.2%	**0.006**	100.0%	95.5%	88.0%	100.0%	**0.083**
**DANGER_R***	9.5%	11.2%	8.8%	**0.405**	7.0%	16.8%	9.4%	6.8%	**0.047**
**FREQ_DEV_R***	93.8%	90.8%	95.2%	**0.065**	94.7%	95.8%	92.1%	93.7%	**0.698**
**DIR_PERS_R***	64.5%	60.5%	66.4%	**0.213**	50.9%	68.4%	64.0%	67.0%	**0.122**
**PAS_FORT_R***	87.6%	82.9%	89.7%	**0.036**	86.0%	85.3%	88.5%	88.5%	**0.837**
**NO_AID_R***	80.5%	76.3%	82.4%	**0.116**	82.5%	82.1%	77.0%	81.7%	**0.668**
**TRAIT_PAS_EFF_R***	75.7%	73.7%	76.7%	**0.478**	71.9%	68.4%	69.8%	84.8%	**0.002**
**Stigmatisation: Probably willing or Definitely willing (≥4)**									
**HAB_MAL**	48.5%	54.6%	45.8%	**0.071**	54.4%	56.8%	38.8%	49.7%	**0.033**
**DISC_MAL**	86.1%	83.6%	87.3%	**0.273**	93.0%	82.1%	85.6%	86.4%	**0.313**
**AMI_MAL**	76.6%	76.3%	76.7%	**0.933**	87.7%	76.8%	70.5%	77.5%	**0.075**
**TRAV_MAL**	67.0%	65.1%	67.9%	**0.551**	71.9%	68.4%	63.3%	67.5%	**0.661**
**MARIA_MAL**	62.7%	67.8%	60.3%	**0.116**	73.7%	74.7%	55.4%	58.6%	**0.004**
**POLI_MAL**	39.2%	48.0%	35.2%	**0.007**	42.1%	45.3%	38.1%	36.1%	**0.477**
**EMB_MAL**	58.9%	57.2%	59.7%	**0.610**	63.2%	55.8%	56.8%	60.7%	**0.725**

*R: reversed score

Table S4. Scores of the scale and the factors: description, reliability, internal consistency and test-retest reliability

	Total Mean (SD)	Range	Cronbach's alpha	McDonald Omega	ICC: IC95% (n=51)
F1	22,08 (2,78)	10-28	0,643	0,872	0,772 IC95%= (0,601; 0,870)
F2	4,56 (1,33)	2-8	0,453	0,625	0,637 IC95%= (0,365; 0,793)
F3	6,35 (1,10)	2-8	0,343	0,666	0,291 IC95%= (-0,242; 0,595)
F4	15,05 (2,77)	5-20	0,608	0,797	0,908 IC95%= (0,838; 0,947)
F5	17,12 (2,40)	8-20	0,595	0,796	0,793 IC95%= (0,637; 0,882)
F6	25,36 (5,15)	11-35	0,815	0,873	0,867 IC95%= (0,7671; 0,924)
Total Scale	90,52 (8,95)	60-115	0,765	0,961	0,869 IC95%= (0,770; 0,925)

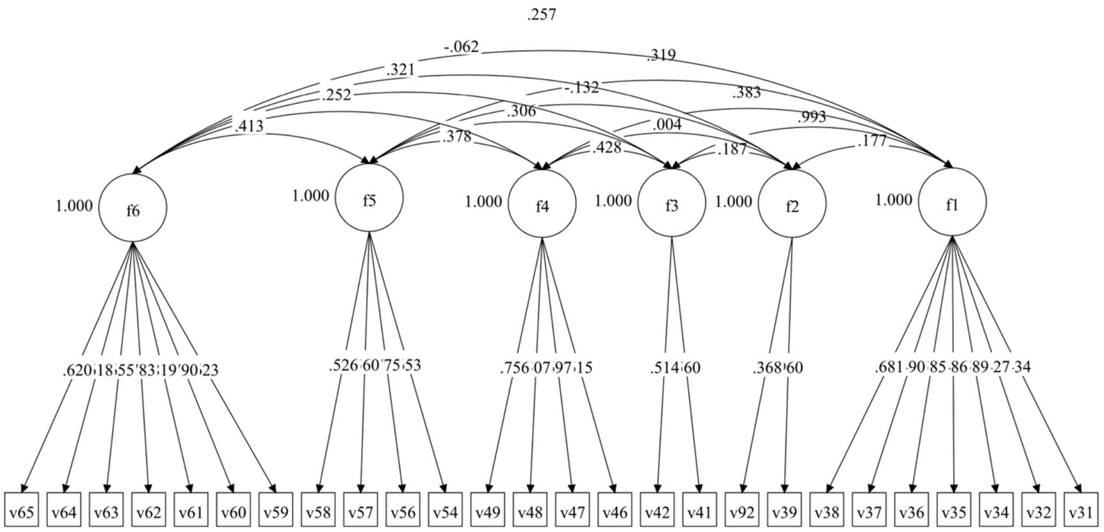

Figure S1. Representation of the confirmatory factor analyses
